# Assessment of Anastomotic Perfusion in Left-Sided Robotic Assisted Colorectal Resection by Indocyanine Green Fluorescence Angiography

**DOI:** 10.1155/2019/3267217

**Published:** 2019-07-14

**Authors:** Emanuel Shapera, Roger W. Hsiung

**Affiliations:** ^1^Surgeon-Resident at Colon and Rectal Clinic of Las Vegas, USA; ^2^Head of Colon and Rectal Clinic of Las Vegas, USA

## Abstract

**Background:**

Indocyanine green fluorescent angiography (IcGA) has been used with success in guiding intraoperative management to prevent colorectal anastomotic complications. Prior studies in open and laparoscopic colorectal surgery, such as PILLAR II, have demonstrated a low anastomotic leak rate (1.4%). As the minimally invasive approach progresses from laparoscopic to robotic approach, the effect and safety of IcGA in assessing anastomotic perfusion in the latter deserve further investigation.

**Methods:**

The objective of the study was to determine the safety of IcGA in guiding intraoperative management of robotic assisted colorectal resection via perfusion assessment. The design was single-surgeon, retrospective case-control study. 74 patients underwent left-sided robotic assisted colorectal resection and anastomosis with IcGA guidance. 30 historical controls underwent left-sided robotic assisted colorectal resection and anastomosis without IcGA. Clinical, demographic, operative, and outcome variables were tabulated.

**Results:**

In the control group, 1 patient suffered a postoperative anastomotic stricture requiring no surgery, and 1 patient suffered an anastomotic dehiscence requiring return to the operating room. There were no anastomotic complications in the IcGA group, including 4 patients who underwent a change in the chosen level of anastomosis based on intraoperative IcGA.

**Conclusion:**

IcGA is safe to use as demonstrated by the very low rate of complications in this case series. It is also safe to rely on to guide re-resection and recreation of an anastomosis intraoperatively by demonstration of blood flow. This may help offset the loss of tactile feedback and assessment of tension in the robotic platform.

## 1. Introduction

Indocyanine green (IcG) fluorescence angiography can assess perfusion of colorectal anastomoses in open and laparoscopic surgery [1, 2]. Its minimal toxicity, near-visible fluorescence (830 nm), and rapid metabolism allow for quick and repeated angiography [3–5].

As colorectal surgery evolves to incorporate the robotic platform, there is a need to study IcGA in this modality where haptic feedback and tactile sensation, critical to assessment of anastomotic integrity, are lacking. Therefore, outcomes for patients undergoing IcG fluorescence angiography (IcGA), including those with intraoperative anastomotic revision, were compared to historical controls without IcGA. This will allow us to assess safety.

## 2. Materials and Methods

104 patients underwent left-sided robotic-assisted colorectal anastomosis by a single colorectal surgeon for various indications. Patient preoperative variables and outcomes were tabulated from a prospectively maintained database between January 2012 and April 2018. Informed consent was obtained from patients. Cases were nonemergent elective resections in patients with established health insurance coverage. A thorough preoperative history and physical was performed to ensure nutritional optimization and lack of frailty. Some LAR cases were done for nonrectal cancer disease due to the extent of resection as guided by IcGA for diverticulitis or prolapse.

**Figure 1:**
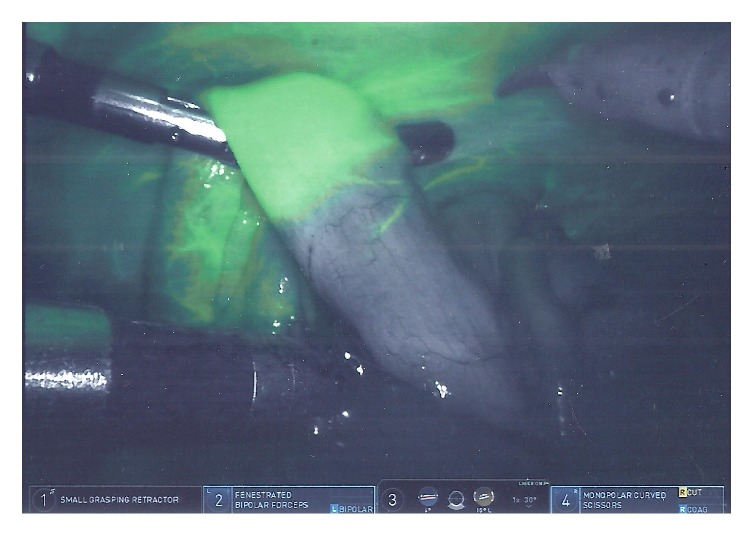
Intraoperative indocyanine green angiography. Change in bowel fluorescence reflects demarcation of vascularized bowel.

A standardized enhanced recovery pathway was faithfully adhered to for all patients, reducing historical bias within the case series. Demographic, clinical, operative, and outcome variables were tabulated (Tables 1–4). All intraoperative decision-making based on IcGA was noted. Anastomoses were performed intracorporeally by an end-to-end stapler. An anastomotic complication was defined as dehiscence or stricture within 30 days, reflecting a disruption of anastomotic integrity or ischemia [6]. IcGA was conducted by mixing 25 mg of indocyanine green with 10 ml of sterile saline. The mixture was injected and the infrared camera activated within 1 minute. Perfusion of bowel would be visualized by the presence or absence of fluorescence (Figure 1). Resection and anastomosis would follow. A second IcGA would then be conducted after the first had been washed out to subjectively determine blood flow around the anastomosis; if this was deemed to be poor, the operating surgeon would resect the anastomosis and select another portion of bowel to utilize. Every attempted indocyanine green angiogram (IcGA) was successful in the study. All cases were done with the da Vinci Xi robot (Intuitive Surgical), which has IcGA capability integrated into the camera with an infrared mode.

## 3. Results

104 patients were included in the final analysis. Median length of stay was 4 days. There were no mortalities. There were two postoperative anastomotic complications in the control group. A 68-year-old female who underwent LAR for diverticulitis suffered a postoperative stricture identified by postoperative colonoscopy at follow-up that did not require any therapeutic procedure. A 54-year-old female with a history of Crohn's colitis, multiple prior abdominal surgeries, and near-obstructing distal sigmoid stricture suffered an anastomotic dehiscence after LAR, lysis of adhesions, and diverting loop ileostomy. Despite the latter, the contamination of her leak resulted in emergent return to the operating due to a necrotizing soft tissue infection. She underwent washout, multiple debridements and end colostomy ultimately surviving to placement in rehab and eventual complete restoration of bowel continuity. Neither of these patients had prior pelvic irradiation.

In the IcGA group, 4 patients underwent an intraoperative change in management based on the subjective assessment of the IcGA fluorescence. This change in management involved selecting a better fluorescing distal segment of bowel to use as part of the anastomosis. The number of IcGA needed to change management was 18. None of these patients suffered an anastomotic complication (stricture or dehiscence).

The postoperative course was uneventful for 84% of patients; 16% had one or more postoperative complications (Table 4). Two of these complications, occurring in a single patient, were Clavien-Dindo Classification grade 4, mandating a single return to the operating room. Otherwise all other complications were Clavien-Dindo Classification grade 1 or 2. Overall mortality was 0%.

## 4. Discussion

There were two anastomotic complications in the control group and were none in the IcGA group, including four patients whose anastomosis was assessed and revised real time intraoperatively via IcGA. Therefore, it is safe to rely on IcGA in assessing and changing the anastomosis via perfusion assessment, fulfilling the objective of our study. Pelvic irradiation occurred more often in the control group; however, its use has not been conclusively shown to impact rectal anastomoses [7–9], and no anastomotic complications occurred in any patient with prior pelvic irradiation in either group.

Anastomotic complications are major contributors to morbidity and mortality in bowel surgery. Prevention relies on tissue integrity and vascularity [10]. There is a paucity of objective measures to gauge these factors [11]. Visible light assessment, including via oxygen saturation spectrometry, is not reliable [12]. Although oxygenation is likely a contributing factor, murine models of bowel anastomoses have conflicting results with hyperbaric oxygen therapy [13, 14]. Assessing pulsatility and mechanical integrity requires tactile feedback which is diminished in laparoscopy and absent in robotic surgery. As the robotic platform is increasingly utilized, IcGA can objectify the assessment of bowel perfusion [11]. Attempts can then be made in operative management to prevent anastomotic complications, as were done in four of our patients successfully.

The benefits of IcGA in laparoscopic LAR have been demonstrated by the PILLAR II study, reporting anastomotic complication rate of 1.4% [15] when it guided anastomotic formation by selecting perfused bowel. Studies in the robotic platform are sparse, despite the fact that this modality provides acceptable oncological and recovery outcomes in LAR [16]. Jafari et al. reported fewer anastomotic complications in robotic LAR cases utilizing IcGA versus historical controls (6 versus 18%) [11]. In their retrospective case series of robotic colorectal anastomoses, Ris et al. demonstrated no anastomotic leaks in their group when IcGA was utilized [17]. This is congruent with our study, which had no anastomotic complications when IcGA was utilized. Studying the effect of IcGA on anastomotic complications via the robotic platform is important as the surgeon, lacking the tactile feedback to assess tension and pulsatility in open or even laparoscopic cases, will rely on IcGA even more.

Additionally, our study corroborates the growing body of evidence supporting the safety of IcGA in revising an anastomosis real time, as demonstrated by Boni et al. in two papers [18, 19], one of which analyzed left-sided resections exclusively.

In contrast, a paper by Kin et al. [20] did not find a difference in over 400 patients. The paper was unfortunately marred by a number of flaws. The investigators did not use IcGA to assess the vascularity of the rectal stump. There was a statistically and clinically significant difference among surgeons in anastomotic leak rate; it is impossible to tell if the more technically challenged surgeons utilized IcGA more often or not.

There are a few limitations that must be highlighted in our study. Our data was matched against historical controls. As with other papers utilizing historical controls, it compares the efforts of the surgeon in a less experienced time point. However the single-surgeon design limits the confounder of technical skill, which can vary significantly among surgeons within a group and account for differences in outcomes. The low rate of anastomotic complications reduces generalizability but occurred in the context of an elective, highly functional patient population. It is one of the largest studies evaluating the effects of IcGA on left-sided robotic assisted colorectal procedures to date and adds to the growing body regarding consequences of real time intraoperative guidance and revision.

IcGA was safe in guiding operative management and has a potential role in reducing anastomotic complications in the robotic platform, where a surgeon may depend on it more exclusively than during laparoscopy or open procedures which have some guidance via tactile feedback and pulsatile assessment. Randomized controlled prospective may validate these findings in the near future.

## Figures and Tables

**Table 1:** Indications for resection.

		IcGA (74)	No IcGA (29)	p value
Malignancy		43	20	1.00

	Rectal	22	14	0.115

	Recto-Sigmoid	6	3	0.715

	Sigmoid	10	2	0.502

	Descending	4	0	0.322

	Splenic	1	1	0.496

Benign Tumor		2	0	1.00

Benign Diagnosis				

	Diverticulitis	22	8	0.815

	Prolapse	7	2	0.433

**Table 2:** Preoperative patient parameters.

	IcGA	No IcGA	p value
*Pre-op Irradiation*	*8*	*9*	*0.0368*
HTN	32	13	1.00
DM II	8	4	0.740
Prior Surgery	31	14	0.669
Female	32	13	1.00
BMI (mean, kg.m^−2^)	27	28	0.431
Age (mean years)	58	60	0.388

**Table 3:** Procedure performed.

	IcGA (74)	No IcGA (30)	p value
LAR	58	23	1.00
Left Hemicolectomy	6	1	0.670
Sigmoidectomy	10	6	0.549

**Table 4:** Postoperative complications.

	IcGA	No IcGA	p value
Ileus	2	3	0.143
Anastomotic dehiscence	0	1	0.289
Post-operative stricture	1	0	0.289
Post-operative bleed	1	0	0.289
Stoma Site Injury	0	1	0.289
Wound Dehiscence	0	1	0.289
Wound Infection	1	1	0.496
Renal Function Impairment	0	2	0.0812
Respiratory Failure	0	1	0.289
Pneumonia	1	3	0.0713
C Diff	1	0	0.289
Urinary Tract Infection	0	2	0.0812
Urinary Retention	3	1	0.0713
Total Complications	10	16	
Patients with complications	9 (12.1%)	8 (26.7%)	0.0839
Total number of complications:			26
Total number of patients with complications:			17 (16%)

## Data Availability

The data used to support the findings of this study are available from the corresponding author upon request.
